# Sparse memory ensembles set brain-wide network states to sustain learned associations

**DOI:** 10.1016/j.isci.2025.113574

**Published:** 2025-09-15

**Authors:** Josué Haubrich, Gabriele Russo, Denise Manahan-Vaughan

**Affiliations:** 1 Medical Faculty, Department of Neurophysiology, Ruhr University Bochum, Bochum, Germany

**Keywords:** Cellular neuroscience, Neuroscience, Systems neuroscience

## Abstract

The ability to recall spatial memories and adapt behavior to changing conditions underlies efficient navigation. These processes are thought to rely on sparse populations of neurons, embedded within distributed brain networks. Here, we sought to address how these neurons impact large-scale network connectivity to sustain behavior. We tagged neurons that were active during an appetitive spatial memory task in TetTag-hM3Dq mice and chemogenetically reactivated these ensembles during fMRI. The reactivation of these ensembles triggered widespread network reorganization, optimizing modular specialization while maintaining integration via key hubs and gateways. We identified distinct clusters dominated by the ventral and dorsal hippocampus that exhibit unique connectivity with cortical and subcortical areas. While control mice showed effective extinction learning (EL), reactivating the tagged memory ensembles prevented EL. These findings reveal brain-wide hubs that support spatial memory and indicate that decay of this network connectivity is likely an essential facet of effective EL of spatial experience.

## Highlights

• DREADD-mediated reactivation of neurons involved in spatial appetitive learning was done
• fMRI and Immunohistochemistry revealed sparse brain-wide networks involved in this process
• Re-activation of the network impairs extinction learning of the previously acquired task
• Suppression of prior memory trace may be a necessary facet of effective extinction learning

## Graphical abstract

**Figure:**
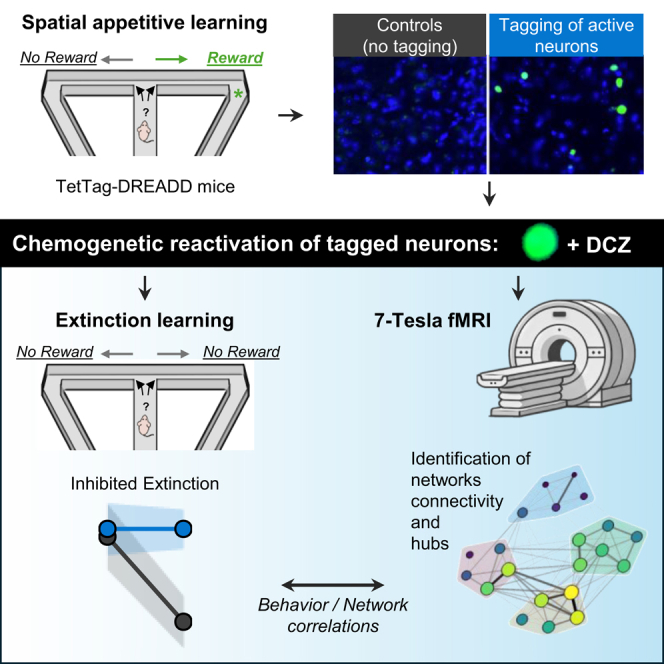

## Introduction

Navigating environments and adapting strategies to changing contingencies rely on activity in specific brain structures, as well as on complex, brain-wide network interactions.^1^^,^^2^ Memories of these learned experiences are encoded by sparse populations of neurons distributed across interconnected circuits.^3^ Although manipulating these sparse memory ensembles within local circuits can trigger or impair the expression of consolidated memories,^4^ it remains unclear how the activity of these ensembles can drive brain-wide information flow and system dynamics.^5^ Here, we investigated how the reactivation of neurons, that were engaged during the retrieval of a well-learned appetitive spatial memory task, influence memory during spatial appetitive extinction learning (EL). We also examined how brain connectivity is represented, by using functional magnetic resonance imaging (fMRI), during this reactivation event.

The dorsal hippocampus is essential for spatial representation and episodic memory,^6^^,^^7^ providing a spatial-temporal framework that enables the creation of a “cognitive map” of the experienced world.^8^ It is particularly critical for encoding trajectories toward rewards,^9^ integrating goal representations within a what-when-where framework,^10^ pattern completion and pattern separation,^11^^,^^12^ as well as supporting memory formation, recall, and updating.^13^^,^^14^^,^^15^^,^^16^^,^^17^ Also essential for effective navigation in a changing world is the ability to recognize that a previous association no longer leads to an expected outcome during EL,^18^^,^^19^ a process that also engages the dorsal hippocampus,^20^^,^^21^^,^^22^ as well as the prefrontal cortex (PFC).^23^

Although it was previously thought to support non-spatial information processing, including anxiety-based behavior,^24^ more recently it has become apparent that the ventral hippocampus also supports spatial learning.^25^^,^^26^^,^^27^ The ventral hippocampus additionally plays a key role in promoting goal-oriented learning and information updating^28^^,^^29^^,^^30^ that are supported through its interconnectivity with the PFC and dorsal hippocampus.^31^^,^^32^

Consolidated associative memories are stored in distributed brain structures beyond the hippocampus,^5^ with interregional interactions supporting encoding and retrieval,^33^^,^^34^ behavioral modulation,^35^ and the updating of predictions during EL.^18^ The retrosplenial cortex (RSP) processes spatial, cognitive, and reinforcement information,^36^ while the cingulate cortex (ACC) supports long-term memory storage^37^^,^^38^ and cognitive control.^39^ The posterior parietal cortex (PPC), by contrast, contributes to multimodal spatial processing and encoding.^40^ In the PFC, the orbitofrontal region (ORB) governs decision-making and association updating,^41^^,^^42^ while the infralimbic (IL) region facilitates decision-making, response adaptation, and EL.^23^^,^^43^ This contrasts with the actions of the prelimbic (PrL) region, which counteracts these processes.^44^^,^^45^

Integral components of brain networks, thalamic nuclei such as the paraventricular (PVT), reuniens (RE), and laterodorsal (LDT) relay sensory information,^46^ support memory functions,^47^^,^^48^ motivate behavior,^49^ direct attention,^50^ and aid spatial navigation.^51^^,^^52^ Moreover, the nucleus accumbens (NAc) is critical for adaptive navigation since it associates environments with salient outcomes^53^ and supports spatial appetitive learning and retrieval.^54^

While spatial navigation and memory involve numerous brain areas, these processes are thought to depend on the activity of sparse neuronal populations within each structure.^3^^,^^55^ In mice, combining long-lasting activity-dependent cell labeling with targeted manipulations has enabled the study of specific neuronal ensembles activated during aversive experience.^56^^,^^57^ This approach allowed gain- and loss-of-function studies to investigate memory-related ensembles across brain regions, including the hippocampus, amygdala, RSP, prefrontal cortex, and the NAc.^34^ While reactivating or silencing tagged memory ensembles can promote or suppress learned behaviors, it remains unclear how the activity of these sparse populations influences brain-wide communication, the extent to which they might be engaged in non-aversive forms of learning, and whether the resulting systemic changes drive behavioral responses.

In this study, we hypothesized that memory-related neuronal ensembles across brain areas influence behavior not only by modulating local circuitry, but also by altering patterns of brain-wide information flow. We also tested the hypothesis that the reactivation of a well-acquired spatial memory, in the absence of a reward incentive, will disrupt EL of this task. In addition, assessed the contribution of neurons within brain-wide networks in this process.

Using TetTag-hM3Dq mice^57^^,^^58^^,^^59^ to label and subsequently reactivate neurons that were engaged during the successful completion of a spatial appetitive learning task, we assessed whether reactivating these neuronal ensembles could sustain learned behaviors without reward. By means of functional magnetic resonance imaging (fMRI) during reactivation, we also examined whether the activity of these neuronal populations alters interregional coordination and functional connectivity across the brain, reflecting the effective acquisition of a spatial appetitive task. Our findings reveal that reactivating these neurons during exposure to the same paradigm in the absence of an appetitive reward prevents successful EL. Neuronal re-activation of memory ensembles revealed hub-like regions and shifts in network state that occur independently of active behavior or conscious recall, and likely reflect the effective acquisition of spatial appetitive memory and resistance to EL.

## Results

### Reactivation of spatial memory ensembles sustains previously acquired behavior during extinction learning

**Figure 1:**
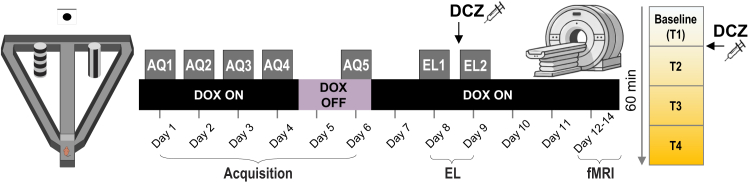
Experimental design TetTag-hM3Dq and Wt mice were trained in a T-maze task (left) to locate a low probability reward based on spatial cues (Acquisition phase: AQ1–AQ5). After four days of training, doxycycline (DOX) was removed to open a tagging window, allowing neurons active during the final training session to express hM3Dq receptors. DOX was reintroduced to the animals’ diet, and two days later, mice underwent an EL session without food reward (EL1). On day 9, a 2nd EL session took place (EL2), where DCZ treatment occurred 30 min beforehand. Functional MRI scanning was performed three to five days later, in the presence of DCZ, to assess brain-wide effects of neuronal reactivation. We performed 60 min of scanning, starting with 15 min of baseline image acquisition (T1), followed by DCZ administration, and continued scanning for an additional 45 min (T2–T4).

We first investigated how the reactivation of a successfully learned spatial appetitive learning task affects EL (Figure 1). TetTag-hM3Dq (TetTag) mice, and their wildtype (Wt) littermates, were maintained on a constant doxycycline (dox) diet. During a 5-day acquisition phase, the mice learned to locate a low-probability reward in a fixed location in a T-maze, relative to spatial cues. One day before the final acquisition session on day 6, dox was removed from the diet to open a window to tag neurons of TetTag mice that were active during this session by means of hM3Dq insertion. The animals then returned to the dox diet, and two days later, on days 8 and 9, underwent a 2-day EL phase. Thirty minutes before the second EL day, the animals received intraperitoneal (i.p.) injections of deschloroclozapine (DCZ) to reactivate the tagged neurons in the TetTag group. The Shapiro-Wilk test indicated that in several sessions, the data was not normally distributed (*p* < 0.05). Therefore, nonparametric statistics were used to detect between-group and within-group differences.

**Figure 2:**
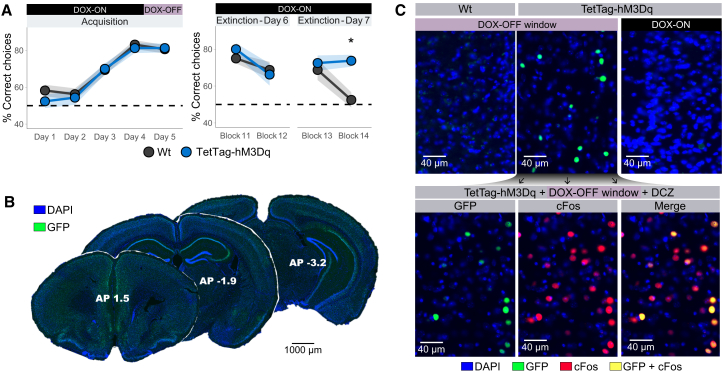
Reactivation of spatial memory ensembles prevents extinction learning (A) Data points represent group means, and the shaded ribbon indicates the standard error of the mean. The upper bars denote the presence (black) or absence (lilac) of doxycycline (DOX) in the animals' diet. *N* = 8 per group, ∗*p* < 0.05, Wilcoxon test. (B) Representative low-magnification images of a TetTag animal after labeling during a dox-off window, showing sparse and widespread GFP-positive neurons. (C) Representative coronal slices from a Wt and TetTag-hM3Dq mice showing tagged cells expressing GFP (green) co-stained with DAPI (blue) following either a dox-off window or a constant dox-on condition (top), and the effect of DCZ on the stimulation of labeled cells, verified by co-expression with cfos (red; bottom).

During the acquisition phase, no differences in performance were observed between TetTag and Wt mice across the training days (Mann-Whitney test, *p* > 0.05). Both groups showed significant improvements in correct choices from day 1 to day 3 and onwards (Wilcoxon signed-rank test, *p* < 0.05). Performance peaked on day 4 and remained stable on day 6 (the day of neuronal tagging) (Wilcoxon signed-rank test, *p* > 0.05) (Figure 2A, left).

During the EL sessions, Wt mice exhibited a significant decrease in correct responses that was evident from the first through the final EL trial block (Wilcoxon signed-rank test, *p* < 0.05). Both TetTag and Wt groups exhibited identical correct choice behavior in the first trial block on day 9 (*p* > 0.05), which was not significantly different from their performance during the last trial block on day 8 (*p* > 0.05). However, the correct choice levels in DCZ-treated TetTag mice remained unchanged in trial block 14, indicating a failure to engage in EL as a result of the activation of the neurons involved in task acquisition (*p* > 0.05). Confirming this interpretation, a significant group difference was observed during the final EL trial block on day 9 (Mann-Whitney test, *p* = 0.003; estimate = −20, CI (confidence intervals) [−30, −10]) (Figure 2A, right).

These results demonstrate that both Wt and TetTag mice developed a preference for the rewarded arm during the acquisition phase, achieving peak performance by the time the “dox-off” tagging window was open. During EL, Wt mice required four blocks of 10 trials to extinguish the learned response. In contrast, the reactivation of tagged neuronal ensembles via DCZ treatment in TetTag mice completely prevented EL. These findings show that the reactivation of neuronal ensembles associated with spatial memory recall is sufficient to sustain learned responses, despite the absence of reinforcement during EL. When neurons not tied to memory were labeled and reactivated during EL, performance was not affected (Figure S1).

Immunohistochemistry was used to verify effective neuronal tagging in TetTag mice, as well as DREADD-mediated neuronal stimulation (see methods). For this, green fluorescent protein (GFP)-expressing neurons were assessed. The dox-off period induced GFP-labeling in TetTag animals but not in Wt animals. TetTag animals, maintained on a constant dox-on diet, displayed no labeling (Figures 2B and 2C top). DCZ treatment reactivated the labeled neurons, as indicated by the co-expression of GFP and cFos (Figure 2C, bottom). In accordance with previous reports,^57^^,^^60^ the dox-off window during acquisition in TetTag animals induced the labeling of sparse neuronal populations throughout the brain (Figure 2B). A histological control indicated that this TetTag-DCZ protocol labels and subsequently reactivates fewer than 10% of DAPI-positive neurons (Figure S2).

### TetTag animals exhibit similar blood-oxygen-level-dependent signal acquisition as in Wt-controls, but different patterns of interregional correlations emerge following the reactivation of spatial memory ensembles

**Figure 3:**
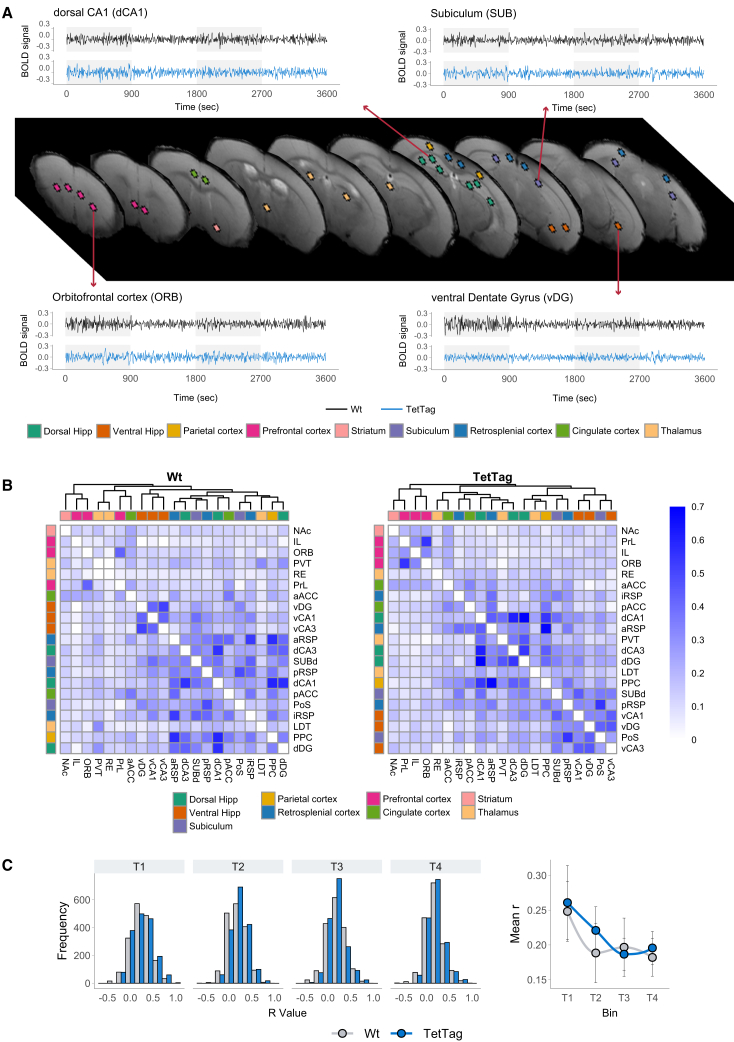
Interregional correlations derived from BOLD signals (A) Locations from which BOLD signals are extracted, and examples of signal time series from a Wt and a TetTag mouse over 60 min of scanning, are shown. (B) Correlation matrices show interregional BOLD signal correlations from the T4 bin for the Wt group (left) and TetTag group (right). Rows and columns are ordered by hierarchical clustering using the complete linkage method. Cells are color-coded according to Spearman’s correlation coefficient values. (C) Left (bar charts): Distribution of Spearman’s correlation coefficients (r values) across time bins. Right (graph): Points denote mean *r* values plotted across bins. Error bars report standard errors of the mean. *N* = 8 animals per group.

To identify brain areas and networks that are engaged during successful spatial memory acquisition, we treated the TetTag mice and their Wt littermates with DCZ during fMRI. BOLD signals were acquired from 21 regions: the dorsal and ventral dentate gyrus (d/vDG), the d/vCA3, and d/vCA1 regions of the cornus ammonis; the dorsal subiculum (SUBd) and the postsubiculum (PoS); nucleus reuniens (RE), laterodorsal (LDT), and paraventricular (PVT) thalamic nuclei; the nucleus accumbens (NAc); the orbitofrontal cortex (ORB); the infralimbic (IL) and prelimbic (PrL) regions of the prefrontal cortex; the anterior, intermediate, and posterior RSP (a/i/p RSP, respectively); the anterior and posterior anterior cingulate cortex (a/p ACC); and the posterior parietal cortex (PPC) (Figure 3A).

To assess the coordinated activity between brain structures, interregional Spearman correlations were calculated for each group and bin (Figure 3B). The analysis focused on T4, the timepoint corresponding to peak DCZ levels in the brain^61^^,^^62^ and the period corresponding to when the behavioral test was conducted (Figure 1B). Reactivation of tagged memory ensembles in TetTag mice revealed interregional clustering patterns that were not evident in control mice (Figure 3B). However, the mean Spearman correlation coefficients across bins were similar between groups (Figure 3C), and no significant between-group differences in correlation values were found at any bin (t-test, *p* > 0.05), nor were there significant within-group changes across bins (paired-sample t-test, *p* > 0.05) (Figure 3C), showing that there were no changes in overall coordinate activity.

These findings indicate that reactivating the spatial memory ensembles that were engaged during memory acquisition does not alter overall levels of interregional correlations when responses in TetTag and control mice are compared. Rather, the reactivation in TetTag mice reveals the coordinated activity between specific brain structures that were presumably engaged during memory acquisition.

### Reactivation of spatial memory ensembles reveals functional network hubs

To further explore the interregional connectivity that emerged when the acquisition ensemble was reactivated, graph networks were constructed based on the computed correlations.^63^ Brain regions were represented as nodes, while significant Spearman correlations between node pairs formed the edges. The weights of these edges corresponded to the correlation coefficients.

**Figure 4:**
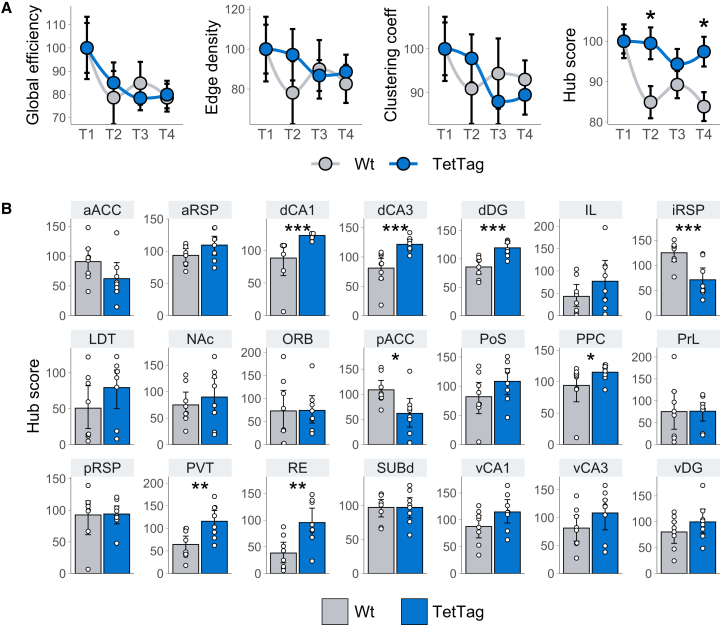
Analysis of network centrality reveals increased Hub regions following reactivation (A) Network-level metrics of global efficiency, edge density, and eigencentrality (Hub score), across time bins and normalized to baseline values (T1). These metrics illustrate changes in network organization following memory ensemble reactivation at the end of T1. Points denote means and error bars the standard error of the mean. (B) Hub score values of individual brain structures in the Wt and TetTag groups, indicating regions with altered network influence after engram reactivation in TetTag-hM3Dq mice. Bars denote means and error bars 95% confidence intervals. Asterisks denote significant differences between groups determined by bootstrapping. ∗*p* < 0.05 (95% CI), ∗∗*p* < 0.01 (99% CI), ∗∗∗*p* < 0.001 (99.9% CI). *N* = 8 animals per group.

To assess the broad effects of spatial memory ensemble reactivation on brain connectivity, network centrality measures were calculated for individual graph, corresponding to each animal and time-bin, and were normalized to each group’s baseline level (T1). We first calculated the network’s global efficiency (the ease of information transfer between nodes),^64^ edge density (the ratio of observed connections to the total possible connections),^65^ and clustering coefficients (the density of triplets within the network).^66^ No significant differences between groups were observed across time points for these metrics (Figure 4A). We then calculated hub scores via eigenvector centrality (also referred as eigencentrality and prestige score), a metric that reflects the importance of a node by considering both its connectivity and the connectivity of its neighbors.^66^^,^^67^ This was used to obtain insights into the emergence of highly influential nodes. The TetTag group exhibited significantly higher average hub scores compared to the Wt group at T2 (95% CI [3.52, 26.1]) and T4 (95% CI [2.33, 23.1]), indicating the formation of more prominent network hubs in TetTag animals (Figure 4A).

To investigate whether specific structures gained prominence following spatial memory ensemble reactivation, hub scores of individual nodes were compared between groups (Figure 4B). Significant increases in hub scores were observed for dorsal hippocampal regions (dCA1, 99.9% CI [14.5, 85.1]; dCA3, 99.9% CI [17.2, 81.3]; and dDG, 99.9% CI [3.21, 58.8]), as well as for associative cortical and thalamic areas (posterior parietal cortex—PPC, 95% CI [2.38, 60.5]; paraventricular nucleus of the thalamus—PVT, 99% CI [9.02, 98.7]; and nucleus reuniens—RE, 99% CI [6.63, 92.8]). Conversely, decreases in hub scores were detected in the intermediate RSP (iRSP, 95% CI [−78.9, 17.6]) and the posterior anterior cingulate cortex (pACC, 95% CI [−79.0, −8.25]).

These findings highlight a shift in the relative influence of specific structures within the network, despite the fact that the overall cohesion of brain connectivity remained unchanged. Reactivation of spatial memory ensembles increased the influence of key nodes, including the dorsal hippocampus, associative cortical regions, and thalamic areas, while reducing the influence of intermediate RSP and posterior anterior cingulate cortices.

### The spatial memory network is governed by a subset of influential structures

**Figure 5:**
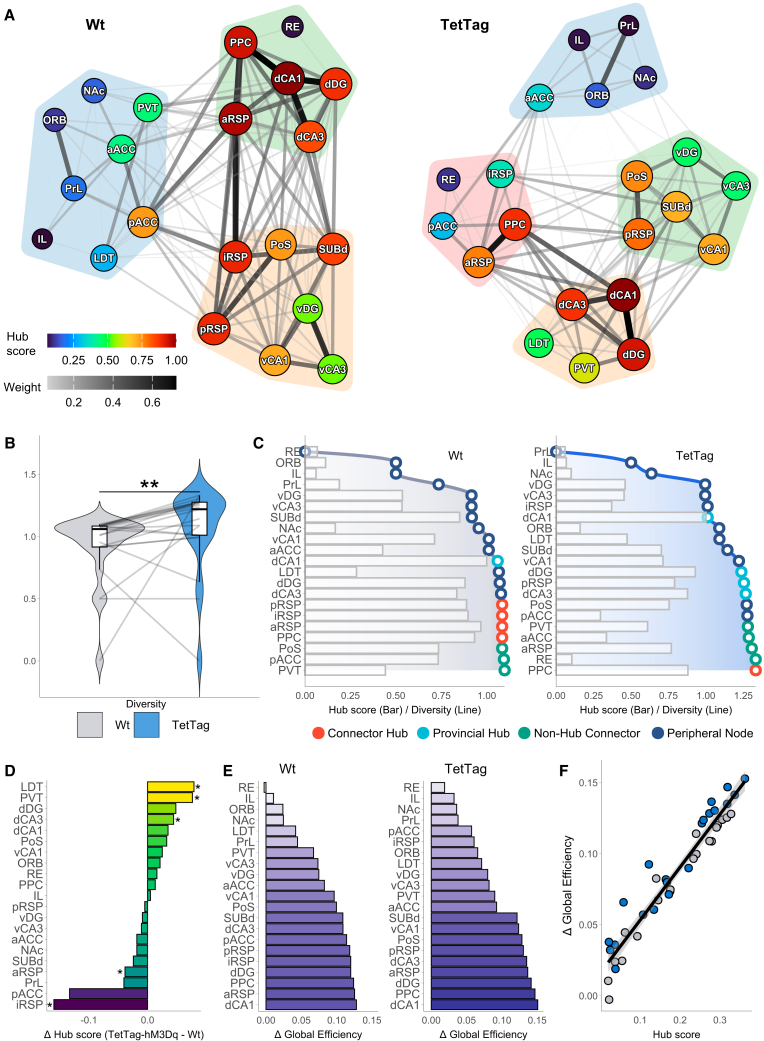
Spatial memory network is modular and governed by a subset of influential structures (A) Graph visualization of the network topology of Wt (left) and TetTag (right) groups. Node hub scores and edge weights are color-scaled. (B) Comparison of connection diversity between groups. Asterisks denote statistical significance obtained using the Mann-Whitney U test, ∗∗*p* < 0.01. (C) Classification of node roles in Wt (left) and TetTag (right) networks based on hub scores (bars) and diversity scores (lines). Points are color-coded to indicate roles: Connector Hubs, Provincial Hubs, Non-Hub Connectors, and Peripheral Nodes. (D) Differences in hub scores between TetTag and Wt nodes. Asterisks indicate statistically significant differences determined by permutation tests (95% CI). (E) Impact of node deletions on global efficiency (ΔGE) assessed using a disruption propagation model in Wt (left) and TetTag (right) networks. (F) Pearson’s correlation between Hub scores and ΔGE. *N* = 8.

To visualize the functional networks of Wt and TetTag mice during peak DCZ efficacy (T4), individual networks were averaged. Correlations that were significant (*p* < 0.001) in at least 75% of the individual networks per group were retained and averaged, ensuring the inclusion of only consistent edges. Communities within these networks were identified using the Louvain algorithm,^68^ with node hub scores represented by fill color and size, and edge weights represented by color and thickness (Figure 5A).

The Wt network was divided into three communities (modularity score = 0.16): a dorsal hippocampus cluster including the RE, PPC, and aRSP; a ventral hippocampal cluster with the subiculum, iRSP and pRSP; and a PFC cluster consisting of the ORB, IL, and PrL, aACC and pACC, nAC, as well as LDT, and PVT of the thalamus (Figure 5A, left). Given that these animals did not undergo ensemble reactivation and were anesthetized, this may reflect resting-state activity.^69^

In contrast, the TetTag network exhibited greater modularity (modularity score = 0.22) with four communities: a dorsal hippocampal cluster including the LDT and PVT, densely connected to a ventral hippocampal cluster containing the subiculum and pRSP; a PFC cluster sparsely connected to the hippocampal clusters, primarily through ORB and NAc; and an additional cluster comprising the pACC, PPC, RE, and a/iRSP, which redistributed PFC inputs to the hippocampal clusters (Figure 5A, right). These differences reveal that the TetTag network displays more localized and modular connectivity compared to controls.

We quantified the diversity of connections in the networks by calculating the Shannon entropy of the communities reached by each node’s neighbors (Figure 5B). This measure reflects how broadly a node connects across different communities. Although the networks did not differ regarding clustering, efficiency, and edge density (Figure 4A), nodes in the TetTag network had significantly higher diversity compared to Wt nodes (Mann-Whitney’s test, *p* = 0.006). This indicates that memory ensemble reactivation enhances cross-community connectivity.

Next, we assessed the diversity and hub score values of individual structures across networks (Figure 5C). Bars represent hub scores, while points indicate diversity values. Within each group, structures were classified based on these scores: those in the top 20% of hub scores were identified as hubs, and those in the top 20% of diversity values were designated as connectors. Structures falling into both categories were classified as Connector Hubs, while those only among the top hub score structures were labeled as Provincial Hubs. Structures not classified as hubs were categorized as Non-Hub Connectors if they scored high in diversity, or otherwise were categorized as Peripheral Nodes. In both networks, dCA1 emerged as the top hub region but was not a connector, indicating its influence is largely confined to the dorsal hippocampal community. In the Wt network, the PPC and all RSP subregions were classified as “Connector Hubs,” while in the TetTag network, only the PPC retained its role as a Connector Hub, with the highest diversity score. The aRSP in TetTag was categorized as a Non-Hub Connector, and other RSP subregions were identified as Peripheral Nodes. Notably, in the TetTag network, non-hub regions such as the PVT, aACC, and RE acted as connectors, while regions such as the dDG, dCA3, and pRSP served as “Provincial Hubs.” In contrast, only dCA1 emerged as a Provincial Hub in the Wt network. Interestingly, the RE ranked last in diversity in the Wt network, but rose to second place during engram reactivation. These changes indicate a sharper division of roles in the engram-reactivation network, likely reflecting an enhanced ability to balance functional specialization within distinct modules with effective inter-community communication. This higher diversity, combined with specialized hubs and connectors, may underlie the network’s capacity to sustain memory-guided behavior.

To highlight differences in node importance induced by memory ensemble reactivation, we calculated differences in hub score between networks (Figure 5D) using a permutation-based approach with network rewiring (10,000 iterations). It revealed significant increases in hub scores for LDT, PVT, and dCA3 compared to controls. Conversely, hub scores decreased in the iRSP and aRSP. We further assessed the importance of individual nodes to network efficiency by simulating node deletions using a disruption propagation model (Figure 5E).^70^ In both groups, the deletion of dCA1 caused the largest drop in global efficiency. However, deletions of pACC and iRSP had a reduced impact in the TetTag network, consistent with hub score differences. While the Wt network showed a gradual change in node deletion impacts, the TetTag network exhibited a clear separation between high-impact and low-impact nodes, further indicating a more specialized network structure.^71^ A strong correlation between hub scores and efficiency impact was observed (Pearson’s *r* = 0.96, *p* < 0.001; Figure 5F), highlighting the predictive power of eigencentrality hub score metric for nodal importance. These results reveal that neurons active during spatial memory retrieval are sufficient to rewire network-wide connectivity patterns related to the functional specialization of individual brain areas.

### Behavior-predictive hubs form a minimal network centered around the dHPC

**Figure 6:**
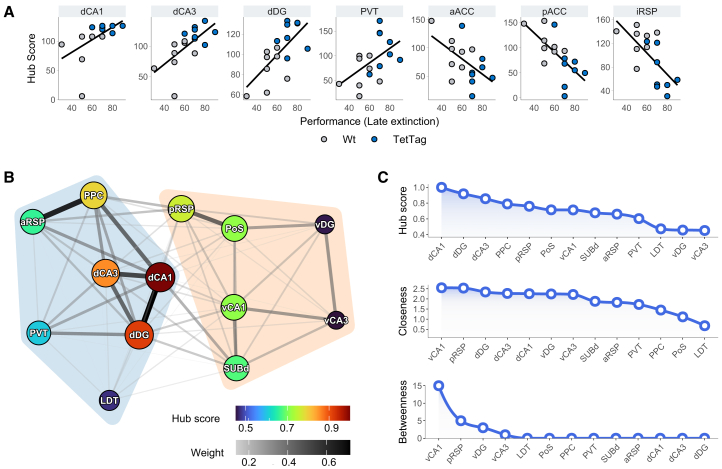
Behavior-associated hubs and minimal memory network (A) Correlation of hub scores with behavioral performance during the last extinction learning block, identifying seven regions with significant Pearson’s correlations. (B) Minimal memory network constructed from regions with positive, significant correlations between hub scores and performance, along with their immediate connections. Node hub scores and edge weights are color-scaled. (C) Rankings of hub scores, closeness centrality, and betweenness centrality values for the minimal memory network.

Although several brain areas increased their network influence following memory ensemble reactivation, not necessarily all were linked to memory retrieval, or task performance. To identify areas whose increased influence was associated with memory recall, we examined the correlation between hub scores and behavioral performance during the late EL phase (Figure 6A). At that stage of the T-Maze task, control animals exhibited reduced correct responses, whereas TetTag mice maintained the learned preference for the previously rewarded maze arm (Figure 2A).

Hub scores of dCA1 (r = 0.52, *p* = 0.034), dCA3 (r = 0.66, *p* = 0.005), dDG (r = 0.68, *p* < 0.005), and PVT (r = 0.52, *p* = 0.04) showed positive Pearson’s correlations with behavioral performance, in accordance with the critical role of these regions in spatial memories. Conversely, aACC (r = −0.55, *p* = 0.03), pACC (r = −0.67, *p* = 0.005) and iRSP (r = −0.67, *p* = 0.005) exhibited negative correlations, consistent with their reduced influence in the TetTag network (Figures 4B and 5B).

To explore the subnetwork associated with memory-guided behavior, we constructed a minimal TetTag network comprising the regions with hub scores positively correlated with late extinction performance and their immediate connections, and communities within these networks were identified using the Louvain algorithm^68^ (Figure 6B). This network revealed two main communities: a dorsal hippocampus-centered community (including dCA1, dCA3, dDG, LDT, PVT, PPC, and aRSP) and a ventral hippocampus-centered community (consisting of vCA1, vCA3, vDG, SUBd, POST, and pRSP). Within this framework, dCA1, dCA3, and dDG emerged as the most influential hubs, driving network integration, while vDG and vCA3 exhibited the lowest influence (Figure 5C).

Notably, despite their lower hub scores compared to dorsal hippocampal regions, vCA1 and pRSP ranked highest in closeness and betweenness centrality, indicating a role in facilitating information flow within and across communities (Figure 6C).

While this minimal network does not capture the full complexity of relationships in the broader network (Figure 5), it highlights the local connectivity patterns of nodes most closely linked to memory-related behavior. Consistent with their complementary functions,^25^^,^^26^^,^^27^ these findings reveal that while the dorsal hippocampus is the minimal network’s core component, it maintains close functional relationships with the ventral hippocampus, relying on critical connectors and modulatory inputs from the subiculum, thalamus, and RSP.

## Discussion

Animals possess the ability to learn rewarded locations and to use spatial cues to efficiently navigate toward them. Equally important is their ability to adaptively modify their strategies during extinction learning (EL) when contingencies change. These memory processes are thought to be mediated by sparse populations of neurons, embedded within interconnected brain circuits.^3^^,^^72^ However, knowledge about how these memory ensembles influence interregional connectivity is limited. To address this, we used TetTag-hM3Dq mice to insert the excitatory hM3Dq receptor into neurons that were activated during appetitive spatial learning, a form of learning that humans rely on daily and is critical for normal functioning. We then chemogenetically reactivated the tagged neuronal ensemble, first during EL and later during fMRI, to examine which brain networks support a successfully learned spatial appetitive task. We demonstrated that the reactivation of neuronal ensembles that were active during successful task acquisition, significantly disrupts EL, given that animals persisted in visiting the target arm, even though a reward was no longer present. At the network level, ensemble reactivation promoted modularity and nodal specialization, reconfiguring hub regions by increasing the importance of some nodes, while reducing that of others. We identified a network “backbone,” closely associated with spatial memory expression, that extends beyond the dorsal hippocampus. This backbone includes ventral CA1, the subiculum and postsubiculum, the posterior parietal cortex, and the retrosplenial cortices, along with the paraventricular and laterodorsal thalamic relays. These findings indicate that neuronal ensembles that are engaged during spatial learning drive goal-directed behaviors by orchestrating connectivity patterns, which guide brain-wide information flow through a cortico-hippocampal-thalamic circuit, the activation of which impedes EL.

Previous studies, using gain- or loss-of-function approaches, have shown that reactivating or silencing neurons that are active during memory encoding, or retrieval, can respectively promote or inhibit learned responses.^73^ These findings have been instrumental in identifying circuits within specific brain structures that are sufficient and/or necessary for various types of memories. For example, neuronal activation or silencing in the hippocampus^60^^,^^74^ and amygdala^75^ have been shown to either enhance or diminish fear-conditioned responses in rodents. In this study, we used TetTag-hM3Dq mice to identify neurons across the brain that were active when animals successfully navigated a T-maze to find a low-probability reward. Tagging was performed at a training stage when animals had already reached peak task acquisition performance, therefore capturing both the retrieval and reinstatement of a well-learned spatial memory, rather than tagging neurons that were involved in *de novo* experience encoding. This strategy revealed the participation of sparse populations of neurons across the brain. We then tested the effect of ensemble reactivation driven by DCZ treatment during EL in the unchanged T-maze context. In mice, successful EL in an AAA paradigm, as used here, typically requires two days of exposure to the unrewarded T-maze.^76^ This timeline was also observed in the control (Wt) group in the present study, in which DCZ had no effect on neuronal ensembles, due to the absence of neuronal tagging and the pharmacological selectivity of DCZ for hM3Dq receptors.^61^ However, when tagged ensembles were reactivated in TetTag mice with DCZ, EL was prevented, and animals continued to show behavior consistent with the task acquired on the acquisition days. These results suggest that the tagged ensembles prompted memory reactivation and perseverance in the previously learned task, though the reward was absent. They also suggest that the reactivation of these ensembles is associated with EL failure. Therefore, although EL is initiated by the recall of the original memory,^14^^,^^18^ our data reveal that artificially sustaining this trace paradoxically blocks EL. The finding implies that EL succeeds only when the initial ensemble reactivation is brief and subsequently suppressed or reorganized, allowing the emergence of alternative activity patterns to encode the new unreinforced contingency. This interpretation aligns with fear-conditioning studies showing that EL suppresses hippocampal fear engram reactivation,^16^ and direct stimulation of fear memory ensembles after EL reinstates fear.^77^ Together, these observations indicate that silencing or remodeling the original engram is a prerequisite for EL across memory valences (appetitive or aversive) and behavioral outputs (goal-directed or passive), highlighting the importance to identify the ensemble properties that must be overcome during EL. To investigate the role of these ensembles upon brain wide function, the tagged neurons were reactivated by DCZ during fMRI. Memory ensemble reactivation did not induce abrupt changes in the overall distribution of, or mean values of interregional correlation coefficients in, the BOLD signal compared to control animals. This overall stability is not unexpected under anesthesia, which is known to suppress neural activity and interregional connectivity.^78^^,^^79^ Moreover, the anesthesia-induced reduction in background activity enhances signal-to-noise ratios,^78^^,^^80^ likely highlighting specific effects driven by hM3Dq receptor activation against a suppressed baseline. Indeed, specific changes emerged between pairs of brain regions. To better understand these alterations, we applied graph theory network analysis.^63^^,^^81^ Metrics such as network efficiency, density, and clustering did not differ significantly following hM3Dq receptor activation, indicating that average properties across the whole network were not affected. However, we observed an increase in average eigenvector centrality values, which reflect the influence of certain nodes within the network.^82^ In other words, specific brain regions became dominant drivers of the networks. Given that higher eigenvector centrality reflects that nodes connect to other nodes that are themselves highly connected,^83^ this indicates the emergence of prominent hub structures that played a role in memory retrieval and reinstatement.

The prominent hub structures identified included the dorsal hippocampus subfields (dDG, dCA3, dCA1), paraventricular (PVT) and reuniens (RE) thalamic nuclei, and posterior parietal association area (PPC). In contrast, the intermediate segment of the RSP (iRSP) and posterior segment of the anterior cingulate cortex (pACC) were less influential. The central influence of the dorsal hippocampus was expected, given its well-established role in memory and navigation.^84^^,^^85^^,^^86^ Similarly, the PPC’s central importance as a connector hub aligns with its involvement in spatial processing.^40^ The RSP, critical for dynamically integrating trajectories within a what-when-where framework,^36^ exhibited a region-specific shift: iRSP had only peripheral network importance, while the anterior and posterior segments acted as connectors and hubs in the network, suggesting functional specialization within the RSP. The PVT and RE also emerged as key players. The PVT, important for relaying homeostatic information to hippocampal and cortical regions,^49^ and the RE, involved in relaying spatial information between the hippocampus and subcortical regions,^87^ both demonstrated network prominence as important connector areas. These connectivity patterns reveal that the activation of neurons engaged in spatial memory retrieval and reinstatement reshapes brain network connectivity, causing a subset of regions to become important hubs. This finding compelled us to further scrutinize how these changes affect network organization and to delineate the specific roles of each region within the network.

The balance between functional segregation and integration across a network is important for maintaining specialized processing within distinct groups of brain regions while also allowing for the exchange of information between them.^88^ For instance, in humans, balanced network integration and segregation are particularly relevant for memory above other cognitive functions.^89^ To explore these dynamics, we built network models and looked for groups of tightly interconnected regions, known as communities, that work together to process information, enabling segregated information processing.^90^^,^^91^ Reactivating memory ensembles increased network modularity, with brain regions clustering into smaller communities, while also broadening cross-community connectivity. This aligns with human fMRI studies demonstrating that, in contrast to a motor task, cross-community connectivity is crucial for memory task performance.^92^^,^^93^ Based on their intra- and cross-community links, we also classified brain areas according to their functional roles in the networks^94^ and found that the TetTag network exhibited a sharper division of roles: more regions were classified as local hubs or non-hub connectors, and fewer as both connectors and hubs, compared to controls. Together, these findings reveal that memory ensemble reactivation is sufficient to promote functional specialization, while maintaining effective inter-community communication through relay regions.

Notably, a network community comprising the pACC, RE, a/iRSP, and PPC emerged as a critical intermediary for information flow between the prefrontal cortex and the dorsal hippocampus. Given the established roles of the ACC,^37^ RE,^51^ RSP,^36^ and PPC^40^ in multimodal sensory integration and memory, the emergence of this community may reflect the integration of mnemonic and spatial signals from the dorsal hippocampus with executive processing from the PFC. Additionally, the LDT and PVT moved closer to the dorsal hippocampal fields compared to controls, in line with their contribution to increased relay of sensory information into the hippocampus during memory acquisition.^46^ On the other hand, the ORB and NAc became the primary gateway linking the PFC and ventral hippocampus. The OFC is known for decision-making and updating associations,^41^^,^^42^^,^^95^ while the NAc links environmental cues with rewarding outcomes.^53^^,^^54^ This mediation may reflect a mechanism by which motivational signals integrate with executive control to drive goal-oriented processing in the ventral hippocampus.

In essence, these findings indicate that reactivating spatial memory neurons reorganizes the brain network around a set of specialized hubs distributed across distinct communities and interconnected by relay regions. To identify the backbone of this core configuration, we applied a disruption propagation model^72^ to systematically delete nodes and thereby assess their impact on network efficiency. While nodal disruptions strongly correlated with hub scores in both groups, in the control network, the effects of node deletion were distributed along a continuum, reflecting a state of low specialization under anesthesia. In contrast, the TetTag network demonstrated a concentrated reliance on a specific group of regions. As expected, the dorsal hippocampal subfields were part of the network backbone. Beyond the well-established importance of the dorsal hippocampus, we also found that the other key members of the network were the vCA1, the subiculum and postsubiculum, the posterior parietal cortex, and the anterior and posterior segments of the retrosplenial cortex. This pattern suggests that information flow across the network is heavily dependent on a dHPC–vCA1–SUB–PoS–PPC–RSP backbone.

The wider network included structures which may reflect the engagement of neurons linked to both pure spatial memory recall and updating (dorsal hippocampus), as well as other functions crucial for goal-directed navigation, such as decision-making (PFC), the processing of sensory cues, and motivation.^96^^,^^97^ To identify regions where network influence was tied to task performance, we correlated hub scores with late EL performance—a period during which only TetTag mice exhibited sustained behavior. Positive correlations were found for dorsal hippocampal subfields and PVT, while negative correlations were observed for ACC and iRSP. These results suggest that increased network influence by the dorsal hippocampal circuit is associated with the behavioral expression of goal-directed, appetitive spatial memory with thalamic participation. To reveal the connectivity patterns driving this outcome, we generated a minimal network containing the dorsal hippocampal regions and their direct connections. The resulting minimal network contained all regions previously identified as forming the TetTag’s network backbone, and additional thalamic structures. Clustering analysis revealed two distinct communities: a dorsal hippocampus-centered cluster and a ventral hippocampus-centered cluster. Within the former cluster, the dCA1-dCA3-dDG subcircuit exhibited the highest network influence, with neighboring nodes including PVT, LDT, aRSP, and PPC. The ventral hippocampal cluster contained SUBd, pRSP, and PoS, with vCA3 and vDG showing the least influence. Although the dorsal hippocampal subfields dominated in terms of hub values, the vCA1 and pRSP ranked highest in closeness and betweenness and were therefore central nodes for transmitting information within and between communities. This suggests that while dorsal hippocampal regions serve as primary hubs, connector nodes, such as vCA1 and pRSP, play crucial roles in maintaining the network’s functional coherence.

Our finding that the ventral hippocampus was involved in spatial memory acquisition is consistent with reports by others.^26^^,^^98^^,^^99^ Interactions of the dorsal and ventral hippocampus during spatial navigation have been demonstrated,^26^ whereby the ventral hippocampus may be especially important for post-learning memory consolidation.^99^ The ventral hippocampus sends afferents to the PFC^100^ and disruption of this communication disrupts cognitive flexibility and spatial working memory.^101^ Successful spatial learning requires bidirectional information flow between the ventral hippocampus and PFC^102^ and spatial information updating requires interaction between the two structures.^32^ Interestingly we did not detect direct connectivity between the ventral hippocampus and the infralimbic and prelimbic cortices during ensemble reactivation, although afferents to these regions have been described.^100^ This may be because these inputs are more relevant to EL^103^ and fear memory.^104^ In addition, when contrasting how influential brain areas were in the TetTag network versus the control network, the thalamic LDT and PVT, along with dCA3, exhibited the most significant positive shifts in network influence upon memory ensembles activation. This likely supported persistent goal-directed behavior during EL, as connectivity among these regions is critical for the mnemonic biasing of attention.^105^

In our network analysis, the reactivation of ensembles tied to an appetitive spatial memory was performed under anesthesia and captured with resting-state fMRI—yet, even in the absence of sensory input, behavior, or conscious recall, large-scale reconfiguration of functional connectivity was observed. These findings demonstrate that internal engram activity alone can drive whole-brain functional reorganization, revealing an intrinsic capacity of memory ensembles to impose specialized network states. This further explains the robust behavioral effects observed here and in prior studies^73^ following engram reactivation, and supports the index-theory^106^^,^^107^ notion that specific neurons serve as a pointer capable of reviving distributed activity patterns.

In conclusion, the results of this study show that sparse, yet broadly distributed, neuronal ensembles that are activated during appetitive spatial memory retrieval are sufficient to reinforce learned responses and inhibit EL. These ensembles drive significant network reorganization, enhancing the brain’s modular specialization while preserving cross-community integration through emergent hubs and gateways. Although the dorsal hippocampus remains central to information flow, effective network communication also critically depends on ventral CA1, the RSP, the PPC, and thalamic nuclei—together forming a core circuit that supports goal-directed navigation and counteracts EL. Furthermore, top-down prefrontal control over the dorsal hippocampus is mediated via a PPC-RSP-RE-ACC circuit, whereas control over the ventral hippocampus implicates the NAc and ORB. In effect, the interference of EL was caused by the emulation of the reactivation of well-established recent memory, indicating that memory network suppression (of the previously learned experience) may be an important facet of effective EL. In sum, we propose that spatial memory-associated ensembles support memory retrieval and reinstatement by inducing widespread changes in interregional communication, channeling information through key hub and modulatory regions that extend beyond the dorsal hippocampus. The dominance of these hubs is sufficient to override EL despite changes in behavioral contingencies.

### Limitations of the study

Our protocol achieved reliable tagging, yet we cannot exclude that alternative dox regimens could enable the labeling of a larger fraction of the original engram. Because tagged neurons were reactivated brain-wide, it remains unclear whether the behavioral persistence observed during EL is driven primarily by memory- or reward-related ensembles, or an interaction of both. Moreover, it is possible that motor circuits were critically involved, but these were not investigated here. Future experiments will be needed to pinpoint their relative contributions. The impact of brain regions upon network function was modeled with *in silico* node deletions. Future studies should test the predictions generated from this approach by performing *in vivo* loss of function experiments (e.g., with chemogenetic or optogenetic silencing). The fMRI assessments were conducted under anesthesia. While responses detected in DCZ-treatedTetTag-hM3Dq mice represent networks engaged by spatial memory, recordings of controls reflected brain activity akin to resting state networks,^78^ and not of networks engaged in active cognitive processes. Future studies could directly compare functional networks driven by spatial memory and EL.

## Resource availability

### Lead contact

Further information and requests for resources and reagents should be directed to and will be fulfilled by the lead contact, Denise Manahan-Vaughan (denise.manahan-vaughan@rub.de).

### Materials availability

This study did not generate new unique reagents.

### Data and code availability

• All data used to make figures available in the main text or the supplementary materials can be found at: https://doi.org/10.5281/zenodo.17177277.
• All code used to make figures and analyses available in the main text or the supplementary materials can be found at: https://doi.org/10.5281/zenodo.17177277.
• Additional raw data are available from the lead contact upon request.

## Acknowledgments

We are grateful to Jens Colitti-Klausnitzer and Juliane Böge for technical assistance with animal treatments, Nadine Kollosch for animal care, Beate Krenzek for genotyping, Dr. Thu-Huong Hoang and assisting student, Rabianur Kartalcik, for support with immunohistochemistry, and Xavier Helluy for assistance with fMRI. This work was supported by a 10.13039/501100001659German Research Foundation (Deutsche Forschungsgemeinschaft, DFG) grant to D.M.-V. (SFB 1280/A04, project number: 316803389). The authors declare no conflict of interest.

## Author contributions

Conceptualization: D.M.V.; data curation: J.H. and G.R.; formal analysis: J.H.; methodology: D.M.V., J.H. and G.R.; investigation: J.H. and G.R.; data interpretation: D.M.V. and J.H.; visualization: J.H., G.R., and D.M.V.; funding acquisition: D.M.V.; project administration: D.M.V.; software: J.H. and G.R.; supervision: D.M.V.; resources: D.M.V.; writing-original draft: J.H. and D.M.V.; writing-review and editing: J.H., G.R., and D.M.V.

## Declaration of interests

The authors declare no competing interests.

## STAR★Methods

### Key resources table

**Table:** 

REAGENT or RESOURCE	SOURCE	IDENTIFIER
**Antibodies**		

Rabbit polyclonal anti-GFP	Invitrogen	RRID: AB_221477
Guinea pig monoclonal recombinant anti-c-fos	Synaptic Systems	RRID: AB_2905595
Goat anti-Rabbit IgG Cy3	Jackson ImmunoResearch Laboratories Inc.	RRID: AB_2338000
Donkey anti-Guinea pig IgG Cy5	Jackson ImmunoResearch Laboratories Inc.	RRID: AB_2340462

**Chemicals, peptides, and recombinant proteins**		

Deschloroclozapine	Tocris	# 7193
Dianova Mounting Medium with DAPI	Biozol Diagnostica Vertrieb GmbH	# SCR-038448
Normal goat serum blocking solution	Biozol Histoprime	# ENG9010

**Critical commercial assays**		

7-Tesla horizontal bore, BioSpec 70/30 USR	Bruker	N/A
MRI CryoProbe	Bruker	N/A
MRI tooth-bar (Z113760) and earplugs (Z113768)	Bruker	N/A
MRI small animal monitoring system	Small Animal Instruments Inc.	N/A

**Deposited data**		

Analyzed data	This paper	github.com/johaubrich
Code for analyses	This paper	github.com/johaubrich

**Experimental models: Organisms/strains**		

Mouse: cfos-tTA/cfos-shEGFP	The Jackson Laboratory	RRID: IMSR_JAX: 018306
Mouse: tetO-hM3Dq	The Jackson Laboratory	RRID: IMSR_JAX: 014093

**Oligonucleotides**		

Primer cfos-tTA/cfos-shEGFP 5’ AAG TTC ATC TGC ACC ACC G 3’	biomers.net GmbH	N/A
Primer tetO-hM3Dq 5’ CGT CAG ATC GCC TGG AGA 3’ and 5’ CGG TGG TAC CGT CTG GAG 3’	biomers.net GmbH	N/A

**Software and algorithms**		

R version 4.2.2	R Foundation for Statistical Computing	https://www.r-project.org/
igraph R package version 2.0.3	igraph	https://igraph.org/
Analysis of Functional Neuroimages (AFNI)	^108^	https://afni.nimh.nih.gov/
Functional Magnetic Resonance Imaging of the Brain (FMRIB) Software Library (FSL v6.0.7)	^109^	https://fsl.fmrib.ox.ac.uk/
Cellpose version 3	^110^	https://www.cellpose.org/
StarDist version 0.8	^111^	
ZEN version 3.1	Carl Zeiss	https://www.zeiss.com/microscopy/de/produkte/software/zeiss-zen.html
QuPath version 0.4.3	^112^	https://qupath.github.io/

### Experimental model and study participant details

The study was carried out in accordance with the European Communities Council Directive of September 22nd, 2010 (2010/63/EU) for care of laboratory animals and all experiments were conducted according to the guidelines of the German Animal Protection Law. The study was approved, in advance, by the North Rhine-Westphalia (NRW) State Authority (Landesamt für Arbeitsschutz, Naturschutz, Umweltschutz und Verbraucherschutz, NRW). All efforts were made to minimize the number of animals used.

#### Mice

We crossed heterozygous TetTag (cfos-tTA/cfos-shEGFP) and tetO-hM3Dq mice (both from The Jackson Laboratory, Bar Harbor, USA) to produce double transgenic TetTag-hM3Dq mice (TetTag group). Control animals were wild-type siblings (Wt group). Polymerase chain reaction (PCR) from ear punch tissue (obtained at <3 postnatal weeks) was used to detect the transgenes. The PCR primers for cfos-tTA/cfos-shEGFP were 5’ AAG TTC ATC TGC ACC ACC G 3’ and 5’TCC TTG AAG ATG GTG GTG CG 3’, and for and tetO-hM3Dq were 5’ CGT CAG ATC GCC TGG AGA 3’ and 5’ CGG TGG TAC CGT CTG GAG 3’ (biomers.net GmbH, Ulm, Germany).

We used 8-12 week-old male (n = 18) and female (n = 12) mice (25-35 g), distributed equally across experimental groups, housed in individual cages, with *ad libitum* access to water, under a 12-hour light/dark cycle with controlled humidity and temperature. Animals were fed with food containing doxycycline (dox; 625 mg/kg; Ssniff, Soest, Germany) and were weighed before commencing the study and subsequently maintained at 85% of their initial body weight. Each mouse was handled daily for at least 5 days before the behavioral procedures. Both groups underwent identical experimental procedures. All experiments took place during the light phase.

### Method details

#### Treatments

Deschloroclozapine (DCZ; Tocris, Bristol, United Kingdom) was freshly prepared on the day of administration by dissolving it in physiological saline. DCZ was administered via intraperitoneal (i.p.) injection at a dose of 0.1 mg/kg. Both Wt and TeTag animals received this treatment.

#### T-Maze protocol

Experiments were conducted in a T-Maze consisting of a starting box made of gray polyvinyl chloride (22 x 10 cm), a central arm (90 x 10 cm), a decision arm (100 x 10 cm), and two return arms (85 x 10 cm), with 19 cm high walls. Distal visual cues placed outside the maze included a cylinder with horizontal stripes to the left, a cylinder with vertical stripes to the right (both made of white cardboard, 50 cm high and 20 cm wide, located 12 cm distally from the respective central arm walls and 15 cm from the inner lateral walls). A white cardboard panel (50 x 50 cm) containing a black circle (25 cm diameter) was positioned 30 cm behind the central arm’s end. A vanilla scent was applied to the end of both decision arms that could only be detected when the animals were at the end of the arms.^113^ The maze was housed in a tent illuminated by white LEDs at 30 lux, and trials were recorded using EthoVision XT v14 (Noldus, Wageningen, The Netherlands).

Mice were pre-handled and subjected to mild food deprivation before training, and their weights were monitored to maintain 85% of their pre-diet weight. Habituation began with 3 daily sessions of 10 minutes in the starting box. This was followed by 6 habituation trials over two days, where mice explored the entire maze. During these trials, they were guided to make a right or left turn without backtracking through the main maze arm, i.e. returning to the starting box via the return arms. Food pellets (Dustless Precision Pellets #F05684, Bio-Serv, San Diego, USA) were scattered throughout the maze to encourage exploration.

The acquisition phase began the following week where mice were placed in the starting box and performed 20 trials per day, to locate a pellet reward that was consistently positioned at the end of the same decision arm. If a preference for one arm was observed during habituation, the reward was placed in the opposite arm. Trials began with the opening of the door to the central arm. Upon reaching the end of a decision arm (left or right), mice were blocked from backtracking and redirected through the return arm to the starting box, marking the end of the trial. During the first three days of acquisition, one pellet was consistently available in the target (rewarded) arm. On acquisition days 4 and 5, the reward probability was progressively reduced from 100% to 40% every five trials, while the reward magnitude was increased from one pellet to four pellets. This approach was employed to increase perseverance in the spatial task enabling gradual, progressive extinction learning (EL). Two days after the final acquisition session, mice underwent an EL session consisting of 20 trials with no rewards. The following day, a final EL session was conducted.

A correct choice was defined as the animal reaching the end of the rewarded arm, during the acquisition phase, whereas turning into the opposite (unrewarded) arm was considered an incorrect choice. If a mouse failed to leave the starting box within 40 seconds after the door to the central arm was opened, this was also counted as an incorrect choice. Similarly, failing to reach the end of a decision arm within 2 minutes was recorded as an incorrect choice. The percentage of correct choices was calculated for each block of 10 trials.

### Experimental design

TetTag mice were maintained on a dox diet to keep the TetTag system inactive. Wt animals received the same diet. The animals underwent training in a spatial appetitive task in the abovementioned T-maze (Figure 1) to learn to locate a reward that was placed with decreasing probability at the end of one specific T-maze arm (Acquisition phase, see above). After four days of training (days 1-4), consisting of two blocks of 10 trials each, and confirmation of successful task acquisition, animals rested undisturbed in their home cages. During this time, dietary dox levels were progressively reduced. After the fourth acquisition session (late morning, Day 4), we halved the dietary dox concentration: the mice received half of their daily ration still containing dox, and ∼6 h later the remaining half without dox. They then spent an entire “rest day” (Day 5) on dox -free chow. The dox -containing diet was provided again only after the fifth acquisition session on Day 6, so the last ingestion of dox -containing food preceded tagging by ∼44 h. This opened a tagging window that allowed highly active neurons - i.e., those with high cFos expression - to express excitatory hM3Dq receptors.^57^ Because wild-type littermates lack the TetTag-hM3Dq transgenes, no tagging occurred in the control group, although controls also followed the same dox diet and dox withdrawal procedures. The efficacy of this tagging procedure was confirmed in immunohistochemical controls experiments (Figures S2, 2B, and 2C).

On day 6, a final acquisition training session was conducted to tag neurons that were active during spatial memory retrieval and dox was reintroduced into the diet immediately afterward (Figure 1). After mice rested for two days in their home cages, EL was tested. We conducted this procedure in the same context (AA paradigm) in the absence of any food reward, a protocol that requires maze exposure on two consecutive days for EL to be effective.^76^ Thus, on day 8, EL sessions were begun, but on day 9, the TetTag animals were systemically injected with DCZ, to reactivate the cells labeled during the acquisition phase of the memory task.^61^ Control (wt) animals also received DCZ treatment: DCZ is a potent, metabolically stable DREADD agonist, with negligible affinity for endogenous receptors, therefore its action is selective to neurons tagged with DREADD receptors.^61^ The EL session on day 9 was commenced 30 min after DCZ administration. Between three and five days later, the mice were anesthetized for assessment using fMRI. After 15 minutes of baseline image acquisition, DCZ was administered, and scanning continued for an additional 45 minutes. (Figure 1).

#### Immunohistochemistry

Immunohistochemistry was conducted post-mortem to verify activity-induced labeling by the TetTag-DREADD system. Animals were maintained on a dox diet. Prior to final acquisition training, one group of TetTag mice had dox removed from their diet, as described above. The other group continued to receive daily dox. Animals then engaged in a further acquisition session in the T-Maze, so that hM3Dq receptors were inserted into neurons that expressed cFos during learning in the dox-free group. Two days later control (wt) and TetTag animals were injected with DCZ and 90 min later, they were perfused transcardially with 4% paraformaldehyde (PFA). Frozen 30 μm sections were prepared using a freezing microtome (Cuttec S, Slee medical GmbH, Nieder-Olm, Germany). Sections were first washed thrice in phosphate buffered saline (PBS) for 10 minutes each. A blocking solution containing 10% normal goat serum (n-Goat; Biozol Histoprime, Germany) and 0.2% PBS-Triton X-100 (PBS-Tx) was then applied, and the sections were incubated, during gentle shaking (Polymax 1040, Heidolph GmbH, Schwabach, Germany), for 90 min at room temperature. A primary antibody solution was prepared, containing rabbit anti-GFP (1:1000; Invitrogen) and guinea pig anti-c-fos (1:2000, Synaptic Systems, Göttingen, Germany) in 1% n-Goat and 0.2% PBS-Tx., Sections were incubated overnight at room temperature during gentle shaking. On the second day, sections were thrice washed in PBS for 10 min each. A secondary antibody solution was prepared, containing goat anti-rabbit Cy3 (1:250; Jackson ImmunoResearch Laboratories Inc., USA) and donkey anti-guinea pig Cy5 (1:500; Jackson ImmunoResearch Laboratories Inc., USA) in 1% n-Goat and 0.2% PBS-Tx, and sections were incubated for 90 min at room temperature with gentle shaking. After incubation, sections were washed thrice with PBS for 10 min each, mounted onto gelatin-coated slides, and left to dry overnight. The following day, slides were briefly rinsed with distilled water and covered with Dianova Mounting Medium with 4’,6-diamidino-2-phenylindole (DAPI) (immunoSelect®; Biozol Diagnostica Vertrieb GmbH, Hamburg, Germany). DAPI-Labeled sections were scanned (Axio SlideScanner) and images were analyzed using imaging software (ZEN , Carl Zeiss, Oberkochen, Germany) or QuPath (v 0.4.3)^112^ to identify neuronal labeling.

#### MRI and fMRI procedures

Imaging was performed using a small animal MRI system (7-Tesla horizontal bore, BioSpec 70/30 USR, Bruker, Germany), equipped with a cryogenically cooled quadrature surface coil for mouse heads (MRI CryoProbe, Bruker, Germany). Mice were anesthetized with isoflurane (3% for induction and 1.5% for maintenance) and stabilized on an animal bed by means of a conventional three-point fixation method that used a tooth-bar and earplugs (Z113760 and Z113768, Bruker, Germany).^114^ Throughout the imaging sessions, body temperature was assessed via a rectal thermometer. A pressure sensitive pad placed underneath the abdomen monitored respiration (Small Animal Instruments Inc., New York, NY, States). Monitoring of blood oxygen saturation and heart rate was conducted with a pulse oximeter clipped to the hind paw (Small Animal Instruments Inc., New York, NY, USA). Cooling and heating were enabled via water pipelines integrated into the mouse bed. Body temperature was maintained at 35.5 ± 1.5 °C during scanning. Imaging data was only collected at blood oxygen saturation values of 97–99%. At the beginning of each imaging session, a fast low angle shot (FLASH) localizer was used to roughly assess the position of the animal’s brain, then images in three different and manually adjusted planes were acquired one after the other using a multi slice FLASH sequence with the following parameters: repetition time (TR) = 522 ms, echo time (TE) = 3.51 ms, no average, acquisition matrix = 120 × 100, field of view (FOV) = 12 x 20 mm, spatial resolution = 0.1 x 0.2 mm^2^, slice thickness = 0.5 mm, number of slices = 16; subsequently, after the measurement of the map shim, a localized shim of the area of interest (whole brain) was performed.

On the basis of the sagittal reference images, 20 coronal slices (Figure S3) were positioned along the entire brain and acquired with single-shot, multi-slice, gradient-recalled echo Planar Imaging (GRE-EPI), with the following parameters: Echo time (TE): 15 ms, TR: 1000 ms, slice thickness: 0.55 mm, no slice gap, matrix size: 95 × 60, FOV: 19 x 12 mm^2^, spatial resolution: 0.2 x 0.2 x 0.55 mm^3^, Bandwidth: 263 kHz, 4500 volumes. The total scan time was 60 min. The first 15 min (T1) served as a baseline period. At the end of T1, all animals received DCZ while remaining inside the MRI system. DCZ was administered intraperitoneally using plastic needles connected to saline-filled tubing, with an air bubble separating the physiological saline and DCZ. Following DCZ administration, scanning continued for an additional 45 min, divided into three bins of 15 min each (T2-T4).

At the end of the experimental session, high-resolution anatomical images, exactly aligned with the functional ones, were acquired using a RARE (**r**apid **a**cquisition with **r**elaxation **e**nhancement) sequence with the following parameters: TR = 2500 ms, effective TE (TEeff) = 15.55 ms, RARE factor = 4, number of averages = 4, FOV = 19 x 12 mm^2^, 20 coronal slices, slice thickness 0.55 mm, no slice gap, matrix size = 190 × 120, spatial resolution = 0.1 × 0.1 mm^2^.

### Quantification and statistical analysis

#### fMRI data pre-processing and time-series extraction

Functional MRI data were processed in their native resolution using a standard pipeline^115^ (Figure S4). First, temporal spikes were removed using analysis of functional neuroimages (AFNI) (https://afni.nimh.nih.gov) 3dDespike. Subsequently, motion correction was performed (3dvolreg). Brain masks (BET, FMRIB Software Library, FSL)^109^ were estimated on temporally averaged echo-planar imaging (EPI) volume (fslmaths). Then, spatial smoothing was applied using an isotropic 0.3 mm kernel (3dBlurInMask) corresponding to 1.5x voxel spacing along the dimension of the lowest resolution. Finally, bandpass filtering (0.01–0.1 Hz) was applied (3dBandpass).

For normalization, the denoised and filtered individual scans were registered to reference space^116^ using corresponding individual anatomical scans to enhance registration accuracy. Regions of interest (ROIs) were defined bilaterally in the reference space as 0.3 mm^3^ spheres. The mean blood oxygen level-dependent (BOLD) signal time-series within a ROI were extracted (fslmeants) and subsequently utilized in the network analysis.

#### Network analysis

We computed Spearman correlations between BOLD signals of all pairs of brain regions acquired during fMRI. Networks were generated by thresholding the interregional correlations for each animal and time bin. Only correlations exhibiting a Spearman p-value of below 0.001, after Bonferroni adjustment for multiple comparisons, were retained. Brain structures were defined as nodes in the network, and the corresponding r-values of the significant correlations served as the weights of the edges. Only positive correlations were considered. Networks were generated for each individual scan and time bin.

For analyses involving averaged networks, we retained correlations that were significant in at least 75% of individual networks within each group. These retained correlations were then averaged and considered as edges in the network. Community detection within these networks was performed using the Louvain algorithm.^68^

#### Centrality measures

Nodal and global network measures were computed using the igraph package in R and custom R code (github.com/johaubrich) to evaluate properties of the functional networks. The metrics included:

- Global efficiency, the average of the inverse shortest path lengths between all pairs of nodes;
- Clustering coefficient, the ratio of the number of triangles to the number of connected triples in the graph;
- Edge density, the ratio of the number of edges present in the graph to the maximum possible number of edges between all pairs of nodes;
- Eigenvector centrality (Hub score), the values of the first eigenvector of the graph adjacency matrix, evaluating a node’s importance by accounting for its connections to other highly influential nodes;
- Betweenness, the number of shortest paths going through a node.

For centrality comparisons that were not based on averaged matrices, measures were averaged per group and time-bin, and expressed as a percentage of the mean value calculated during the baseline (T1). Differences in centrality measures between groups were compared by resampling the data with replacement 1,000 times, to generate bootstrap distributions of the mean differences between groups for each measure and time bin. We calculated 95%, 99%, and 99.9% confidence intervals using the bias-corrected and accelerated (BCa) method. Significance was determined based on whether these confidence intervals excluded zero.

We calculated the diversity score based on Shannon entropy^117^ to assess how many distinct communities were reached by each node’s neighbors. For each node, the communities of its neighboring nodes were identified, and the probability distribution of these communities was computed. The Shannon entropy of this distribution quantified “community diversity,” with higher values indicating that a node connects to a broader array of communities. Diversity scores were compared using the Wilcoxon test.

The Hub score was calculated by subtracting the nodal eigenvector centrality values of the Wt group from those of the TetTag group. To assess the significance of these differences, we performed a permutation-based test using network rewiring, generating null models through 1,000 iterations. P-Values were computed by determining the proportion of times the absolute value of the differences from the rewired networks was greater than or equal to the absolute value of the observed difference.

To evaluate the effect of removing a node on the network’s global efficiency (GE), we computed the global efficiency of the network before node deletion minus its efficiency after deletion. Considering that brain inactivation has functional effects spreading to other connected regions,^118^^,^^119^ we employed a previously described disruption propagation model.^70^

#### Statistics

For normally distributed data, which passed the Shapiro-Wilk test of normality, differences between groups were tested using the Student’s t-test. For non-normally distributed data, we employed the Wilcoxon test, bootstrapping, or permutation non-parametric approaches, as described above. Except for the generation of interregional correlation matrices, all other correlations were conducted using Pearson’s correlation test. All statistical analyses and plots were generated using R version 4.2.2 (R Foundation for Statistical Computing, Vienna, Austria).
