# Supplementary material for: Sparse memory ensembles set brain-wide network states to sustain learned associations
Source: iScience. 2025 Sep 15;28(10):113574. doi: 10.1016/j.isci.2025.113574 (PMC12514527; doi:10.1016/j.isci.2025.113574)
Supplement: Document S1. Figures S1–S4 [file mmc1.pdf]

iScience, Volume *28*

Supplemental information

Sparse memory ensembles set brain-wide network states to sustain learned associations

Josué Haubrich, Gabriele Russo, and Denise Manahan-Vaughan

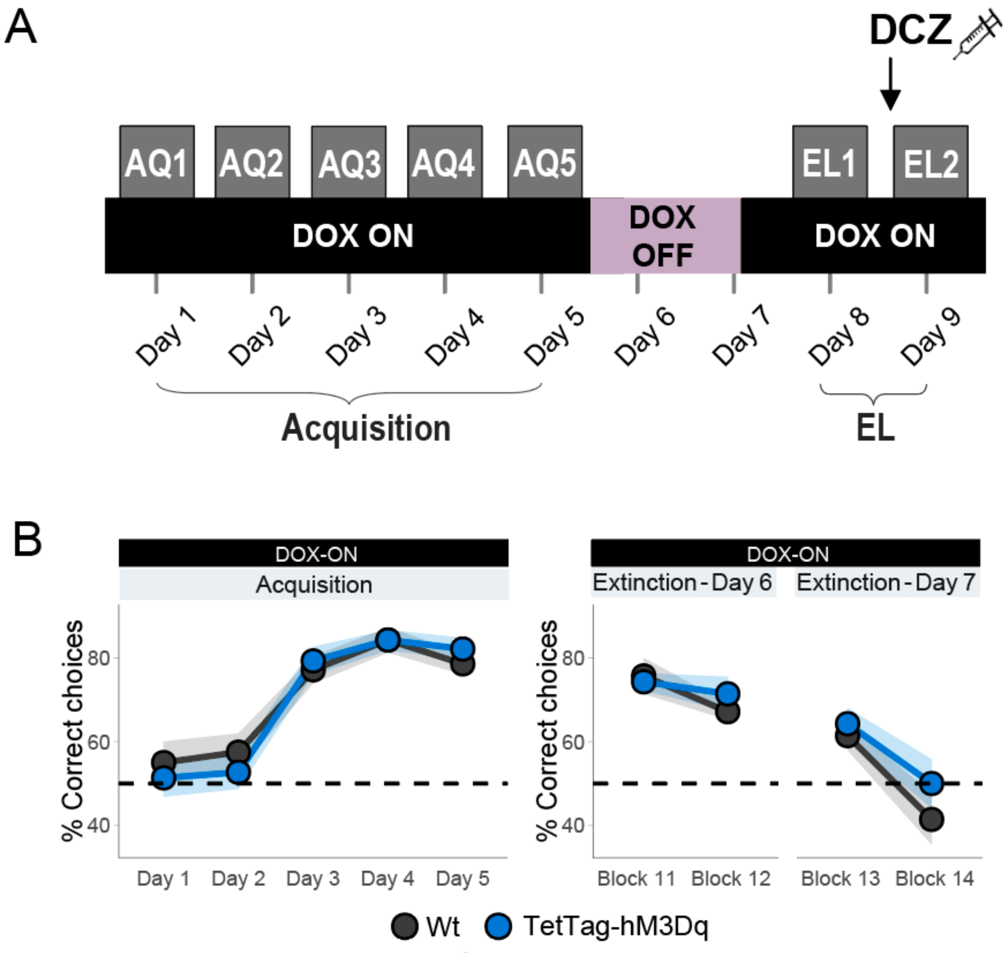

Supplementary Figure 1

Assessment of ensemble activation in the absence of task-related learning

To assess the specificity of the effects observed when tagging neurons active during memory acquisition, A) we repeated the experimental design from Figure 2A but tagged neurons in a home cage condition instead, therefore labelling neurons not tied to the memory task. On the last day of acquisition (Day 5), the doxycycline (dox) concentration in the diet was halved. On the following day (Day 6), dox was absent from the diet, and reintroduced at the end of Day 7. The animals remained undisturbed in their home cages from day 6 to 7. Two days later, the extinction phase began, and animals received DCZ 30 minutes before the second extinction learning (EL) session to reactivate the tagged neurons. This procedure was conducted in both wild-type (Wt) and TetTag-DREADD animals. B) As in Figure 2A, no differences were observed between Wt and TetTag-DREADD mice across the acquisition days (left; Mann-Whitney test, $p > 0.05$). Both groups showed significant improvement in correct choices from Day 1 to Day 3 and beyond (Wilcoxon signed-rank test, $p < 0.05$), with performance peaking on Day 3 and remaining stable through Day 5 (Wilcoxon signed-rank test, $p > 0.05$). During the EL sessions (right), both groups exhibited a significant decrease in correct responses from the first to the final trial block (Wilcoxon signed-rank test, $p < 0.05$), with no differences between groups at any block (Mann-Whitney test, $p > 0.05$). Data points represent group means, and shaded ribbons indicate the standard error of the mean. The upper bars show the presence (black) or absence (lilac) of doxycycline in the animals' diet. N = 7 per group.

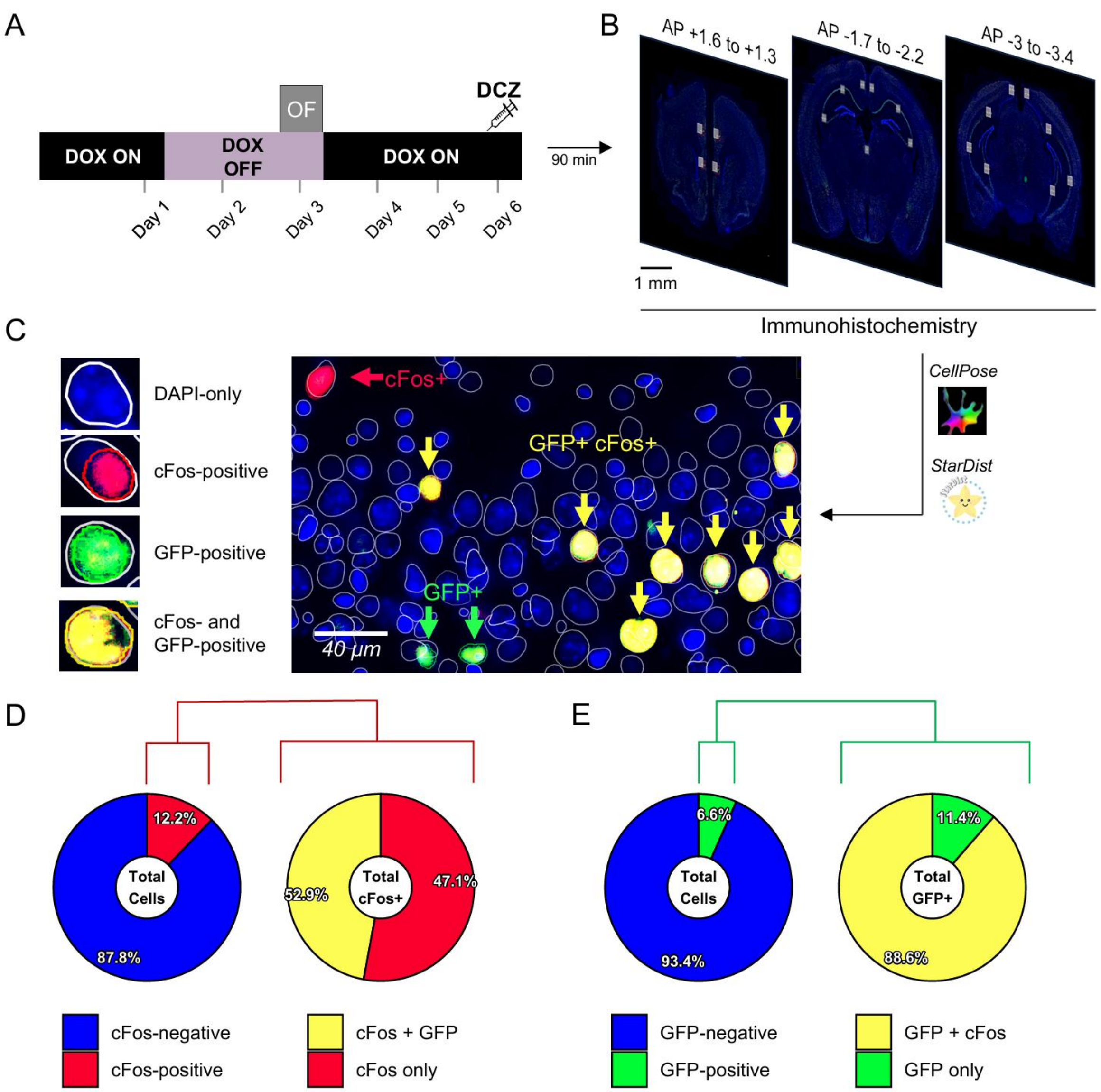

Supplementary Figure 2

Histological assessments of TetTag labeling

A) To assess the extent of neuronal labeling achieved using the TetTag-DREADD system under our doxycycline (dox) protocol, three TetTag-DREADD mice maintained on a dox diet underwent gradual dox removal: the concentration was halved on Day 1 and fully withdrawn on Day 2, creating a labeling window on Day 3. During this window, mice were exposed for 5 minutes to a 90 × 90 cm open field (OF) arena. Immediately after this exposure, dox was reintroduced to close the labeling window. Three days later, animals received an intraperitoneal injection of DCZ and were perfused 90 minutes later for immunohistochemistry (IHC). **B**) Labeled neurons were quantified in 300 x 300 µm regions of interest (ROIs) across ten bilateral brain regions (PL, IL, aRSP, pRSP, dCA1, dCA3, PV, vCA1, vCA3, SUB), using 2–3 slices per animal. **C**) Cell detection was performed using the CellPose[107] algorithm (cyto3 pretrained model) for detecting GFP and cFos positive nuclei, and StarDist[108] for detecting DAPI, both in QuPath[109]. **D-E**) Mean cell counts were first computed per region within each animal. These ten regional means were then averaged to yield a single value per animal, and the animal means were pooled to generate the group mean ± SEM: a sparse fraction of DAPI-positive nuclei was cFos-positive (D, left; 12.2 ± 0.75%) or GFP-positive (1E, left; 6.61 ± 0.97%), and approximately half of the cFos-positive cells co-expressed GFP (D, right; 47.1 ± 4.1%), whereas the majority of GFP-positive cells co-expressed cFos (E, right; 88.7 ± 4.3%). Pie charts display means across animals.

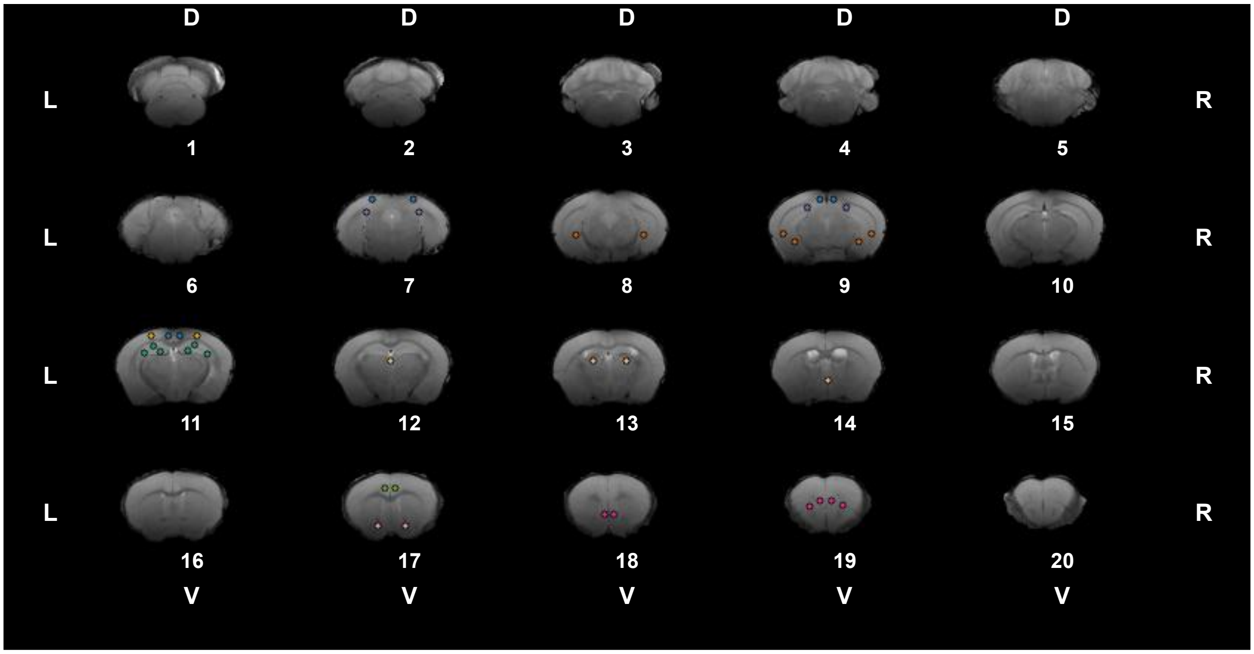

Supplementary Figure 3

Coronal slices processed during fMRI.
L, R, D, and V denote the left, right, dorsal, and ventral directions, respectively. Slices are numbered from posterior to anterior. Seeds are marked with colored circles.

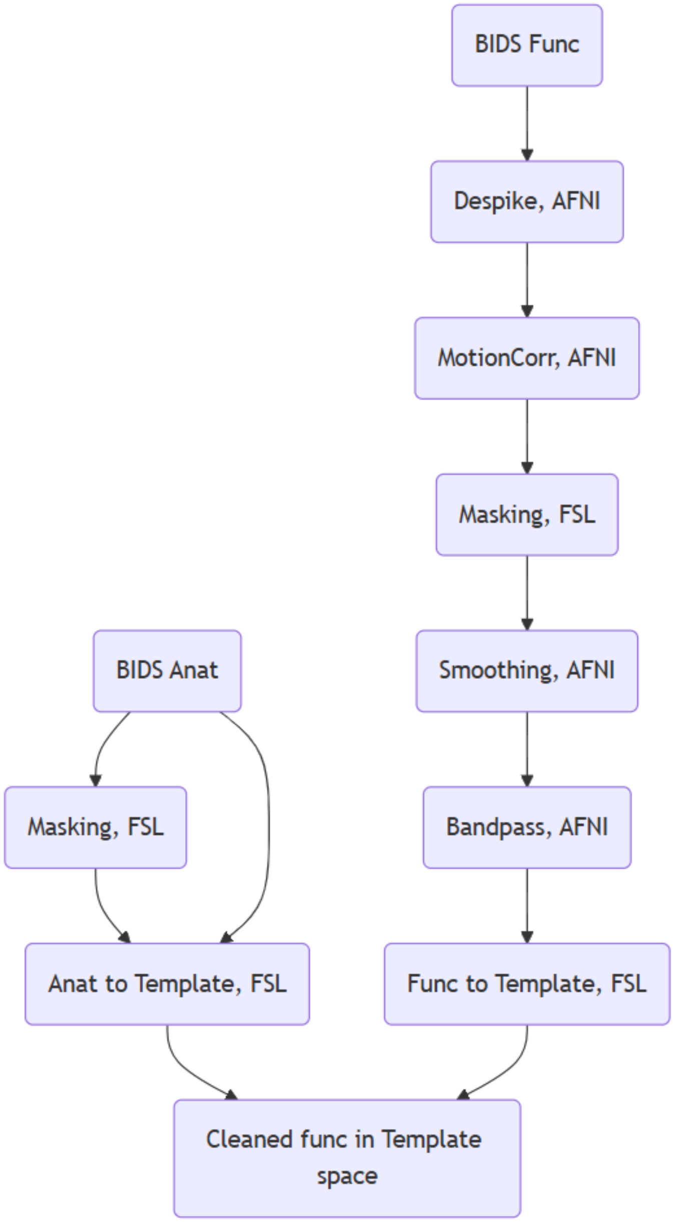

Supplementary Figure 4

Functional magnetic resonance imaging (fMRI) pipeline

Diagram of the fMRI pipeline for data pre-processing and time-series extraction.

Abbreviations: AFNI: Analysis of Functional NeuroImages; Anat: anatomy; BIDS: brain imaging data structure; Despike: truncation of large spikes in the fMRI times series; FSL: FMRIB Software Library; func: functional image; MotionCorr: motion correction.
